# Neutrophil–Lymphocytes Ratio as Potential Early Marker for Alzheimer's Disease

**DOI:** 10.1155/2024/6640130

**Published:** 2024-06-30

**Authors:** Carlo Cervellati, Dario Pedrini, Pietro Pirro, Paola Guindani, Carlo Renzini, Gloria Brombo, Giovanni Zuliani

**Affiliations:** ^1^ Department of Translational Medicine and for Romagna University of Ferrara, Via Luigi Borsari 46, Ferrara 44121, Italy; ^2^ Associazione Sammarinese di Geriatria e Gerontologia (ASGG), Dogana, San Marino

## Abstract

**Background:**

Neutrophil–lymphocyte ratio (NLR) is a noninvasive, inexpensive, and easily applicable marker of inflammation. Since immune dysregulation leading to inflammation is regarded as a hallmark of dementia, in particular Alzheimer's disease (AD), we decided to investigate the potentials of NLR as a diagnostic and predictive biomarker in this clinical setting.

**Materials and Methods:**

NLR was measured in the blood of patients with AD (*n* = 103), amnestic type mild cognitive impairment (aMCI, *n* = 212), vascular dementia (VAD, *n* = 34), and cognitively healthy Controls (*n* = 61). One hundred twelve MCI patients underwent a regular clinical follow-up. Over a 36-months median follow-up, 80 remained stable, while 32 progressed to overt dementia.

**Results:**

NLR was higher in patients with aMCI or dementia compared to Controls; however, the difference was statistically significant only for aMCI (+13%, *p*=0.04) and AD (+20%, *p*=0.03). These results were confirmed by multivariate logistic analysis, which showed that high NLR was associated with an increase in the likelihood of receiving a diagnosis of aMCI (odd ratio (OR): 2.58, 95% confidence interval (CI): 1.36–4.89) or AD (OR: 3.13, 95%CI: 1.47–6.70), but not of VAD. NLR did not differ when comparing stable vs. progressing aMCI.

**Conclusions:**

This is the first report showing that NLR is significantly increased in MCI and AD but not in VAD. We also found that NLR was unable to predict the conversion from aMCI to AD. Further research on larger cohorts is warranted to definitely ascertain the application of NLR as a possible marker for aMCI and AD.

## 1. Introduction

Alzheimer's disease (AD) is the most common cause of dementia and one of the leading causes of morbidity and mortality in the older population [1]. Other frequent types of dementia are, in decreasing order of frequency, vascular dementia (VAD), Lewy body disease, and frontotemporal dementias [2]. All types of dementia are preceded by a preclinical phase, which may also be unrecognized, called mild cognitive impairment (MCI) [3]. From the stage of MCI, the patients can subsequently progress to the established form of the disease; in particular, around 15% of MCI patients convert to AD every year [4].

AD is a multifactorial and heterogeneous disease, the pathogenesis of which has not yet been fully elucidated. Amyloid-beta (A*β*) plaques and neurofibrillary tangles in AD brain are widely regarded as the two typical and specific physiopathological traits of the disease [5]. However, over the years, other pathogenetic mechanisms have been identified in AD, such as vascular dysfunction, oxidative stress, and neuroinflammation; the latter has gained the definition of a real pathological hallmark of the disease [6, 7, 8, 9]. Microglia and, at minor extent, astrocytes are the major players in neuroinflammation [6]. They are primarily involved in host immune responses in the CNS. In particular, microglia are able to eliminate pathogens and attenuate neuronal damage through different mechanisms, including phagocytosis, generation of free radicals, secretion of cytokines/chemokines, and acute phase proteins [10]. In AD, these cells are hyperactivated, and this state is associated with an exaggerated and uncontrolled inflammatory response, which, in turn, may contribute to neurodegeneration [11]. As proof of concept, chronic hyperactivation of these cells has been observed in AD brain and is correlated with A*β* burden and cognitive decline [12]. Moreover, there is solid evidence showing that variants of TREM2 and CD33, microglia receptors, increase the risk for late-onset AD [13, 14].

A wealth of preclinical and clinical evidence suggests that peripheral immune cells can influence the course of neuroinflammation in AD [6]. The cross-talk between systemic circulation and CNS can take place through many routes, including the migration of blood-borne immune cells and the entrance of peripheral cytokines into the brain [6, 9, 15]. Cytokines secreted by blood neutrophils and lymphocytes seem to be involved in the activation of microglia [6, 15]; moreover, increased plasma levels of cytokines have been reported in AD patients [16, 17].

One available clinical marker of peripheral inflammation is the neutrophil-lymphocyte ratio (NLR) [18, 19]. Higher values of NLR have been related with a high risk and poor prognosis of various diseases, such as cancer and coronary heart disease (CHD) [18, 20, 21]. Despite the aforementioned literature supports the existence of a link between systemic inflammation and dementia, this topic has been seldom investigated.

We evaluated whether an alteration in NLR might be a specific and early feature of AD by both cross-sectional and longitudinal approaches. To this aim, we compared the NLR values of cognitively healthy controls with those of patients affected by amnestic MCI (aMCI), AD, and VAD. Moreover, we investigated whether baseline levels of NLR might be predictive in the clinical progression from MCI to dementia.

## 2. Patients and Methods

### 2.1. Subjects

From a sample of 456 consecutive older subjects (>65 years) referred to the Center for Cognitive Decline and Dementia (CDCD) of the University Internal Medicine, Arcispedale S. Anna, Ferrara, Italy, 456 individuals were enrolled into the present study:

1. One hundred three patients with mild–moderate probable AD by the National Institute on Aging–Alzheimer's Association (NIA–AA) criteria [22].
2. Two hundred fourteen patients with amnestic MCI are defined as those with a presence of either short or long-term memory impairment, with or without impairment in other single or multiple cognitive domains [23]. Of these patients, 112 underwent a regular clinical follow-up after the first visit at the CDCD as outpatients. Over a 36-months median follow-up, 80 remained stable, while 32 converted to AD.
3. Thirty-four patients with VAD were diagnosed by the NINDS–AIREN criteria [24]. Only patients with “probable” VAD were enrolled.
4. Sixty-one subjects, without any evidence of cognitive decline and/or functional disabilities attributable to cognitive impairment, served as controls (Controls).

Personal data and anamnesis were collected by a structured interview from patients/caregivers at the first visit. General and neuropsychological examinations, including standardized Mini-Mental State Examination (MMSE [25], upon permission from PAR was granted to use this scale), geriatric depression scale, and CDR, were carried out as previously described [26]. The functional status was evaluated by basic and instrumental activities of daily living (BADL and IADL, respectively) [27]. Demographic and medical history data (e.g., hypertension, CHD, stroke, diabetes, chronic obstructive pulmonary disease) were collected by trained personnel [27].

Subjects with missed or incomplete clinical/demographic data, severe liver or kidney disease, congestive heart failure or chronic obstructive pulmonary disease, cancer, acute illnesses, or using nonsteroidal anti-inflammatory drugs, antibiotics, or steroids at the time of recruitment were excluded.

All subjects underwent a brain MRI and/or an 18F-FDG PET. The presence of leukoaraiosis, lacunar strokes, cortical/sub-cortical strokes, and brain atrophy were evaluated based on MRI.

Criteria used for the diagnosis of diabetes, hypertension, CHD, stroke, and smoking classification (greater than or equal to 10 pack-year history of ever smoking) were described in detail elsewhere [26].

The study was carried out in accordance with the guidelines provided by the Declaration of Helsinki (World Medical Association, https://www.wma.net), and it was approved by the Local Ethic Committee of Arcispedale S. Anna, Ferrara (protocol no. 170579). Signed informed consent, written in compliance with local and national ethical guidelines, was obtained from each patient before the inclusion into the study. All participants (and their caregivers, if demented) were informed about the research project and provided written consent.

### 2.2. Routine Blood Testing

Clinical chemistry analyses were routinely performed to check the health status of the patients as well as to exclude causes of secondary cognitive impairment. Fasting blood samples were obtained via sterile venipuncture (ethylenediaminetetraacetic acid, EDTA was used as an anticoagulant). Routine plasm parameters were analyzed using the standard laboratory method and they included serum B12 vitamin, glucose, folate, lipid profile, C-reactive protein, liver, kidney, and thyroid function tests, and arterial oxygen saturation. Blood cell count was evaluated by an automatic hematology analyzer (Coulter LH 750; Beckman Coulter Inc., United States). The NLR was calculated from the differential count by dividing the absolute neutrophil count by the absolute lymphocyte count.

### 2.3. Statistical Analysis

Differences between sample groups were evaluated by ANOVA (Bonferroni as post hoc test for pair-wise comparisons) or Kruskall–Wallis (Wilcoxon–Mann–Whitney test followed by Bonferroni adjustment) for not normal distributed data. Prevalence was compared by the *χ*^2^-test. The risk of receiving a diagnosis of MCI, AD, VAD in subjects with high values (over the median value calculated in the whole sample) of NLR was assessed by univariate and multivariate logistic regression analysis. The set of covariates to include in the multivariate model was selected on the basis of two criteria: (1) well-known risk factors for dementia, such as age, sex, smoking, and comorbidities, and (2) correlates of NLR. Finally, the association between NLR and other continuous variables was assessed by Pearson's or Spearman's correlation, depending on their distribution. The independence of these associations was checked by multiple regression analysis.

A two-tailed probability value <0.05 was considered statistically significant. SPSS 17.00 for Windows (Chicago, IL, USA) was used for statistical analysis.

## 3. Results

### 3.1. General Characteristics of the Sample

As shown in Table 1, the 456 subjects had similar ages, with VAD patients slightly being older than Controls (*p*  < 0.05). No significant differences across the diagnostic groups were detected as regards sex and smoking prevalence. The frequency of CHD, diabetes, and history of stroke was similar in the four groups. Hypertension was more prevalent in aMCI and VAD compared with AD (*p*  < 0.05). As expected by selection criteria, the MMSE score was lower in all dementia groups compared to Controls (*p*  < 0.01) and aMCI (*p*  < 0.05). No significant changes were detected for any of the biochemical parameters assessed in the study.

### 3.2. Possible Association between NLR and MCI or Dementia: Cross-Sectional and Longitudinal Analysis

**Figure 1:**
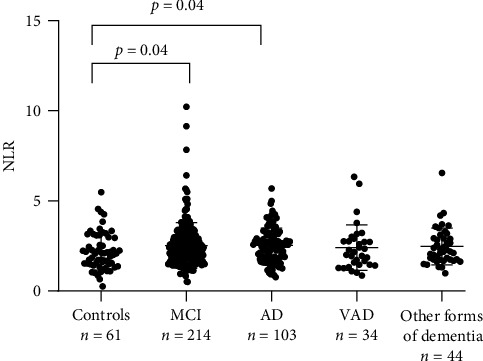
NLR in Controls, mild cognitive impairment (aMCI), Alzheimer's disease (AD), and vascular dementia (VAD).

Compared with Controls, the trend for neutrophil and lymphocyte counts was increasing and decreasing, respectively, in both aMCI and both dementia groups (Table 1). Of consequence, the NLR value was generally higher in patients with aMCI or dementia; however, the difference was statistically significant only for aMCI (+13%, *p*=0.04) and AD (+20%, *p*=0.03) compared with Controls (Table 1 and Figure 1).

**Figure 2:**
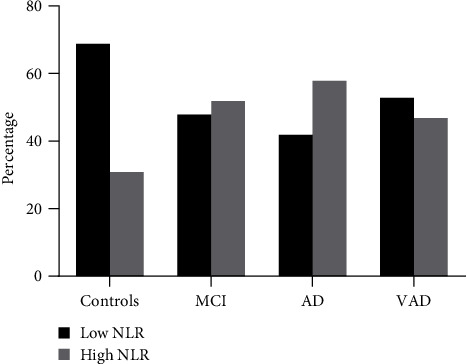
Percentage of individuals with high or low NLR among Controls, mild cognitive impairment (aMCI), Alzheimer's disease (AD), and vascular dementia (VAD).

**Figure 3:**
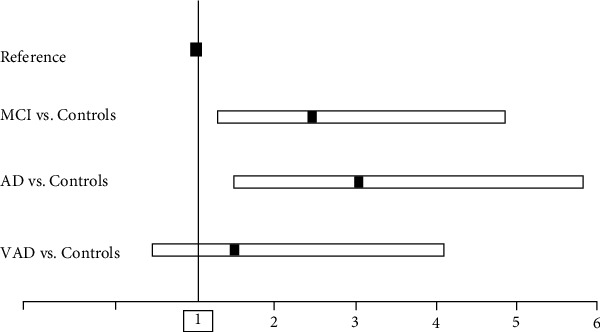
Multi-adjusted odds ratio (95% confidence interval) for the diagnosis of mild cognitive impairment (aMCI), Alzheimer's disease (AD), or vascular dementia (VAD) in subjects with high levels (above the median value: 2.276) of NLR. Covariates: age, gender, hypertension, CHD, stroke, diabetes, smoking status, albumin and triglycerides.

We also calculated the percentage of individuals with NLR higher/lower than the median value (2.276). The prevalence of subjects with “high” NLR was near or above 50% in aMCI, AD, and VAD (52%, 58%, and 47%, respectively), while it was 31% in Controls (Figure 2). These results, along with the absence of an established cutoff for this metric, prompted us to use the median value to dichotomize NLR as an explanatory variable in logistic regression analysis. It emerged that, compared to Controls, “high” NLR was associated with an increase in the likelihood of receiving a diagnosis of aMCI (odd ratio (OR): 2.40, 95% confidence interval (CI): 1.31–4.41) or AD (OR: 3.03, 95%CI: 1.55–5.92). On the contrary, there was no association with VAD (OR: 1.96, 95CI%: 0.82–4.66). As shown in Figure 3, the odds of having aMCI or AD in individuals with high NLR were still significant for aMCI (OR: 2.58, 95%CI: 1.36–4.89) and AD (OR: 3.13, 95%CI: 1.47–6.70), after multivariate adjustment for potential confounders, including age, sex, smoking, comorbidities, albumin (NLR vs. albumin: *r* = −0.100, *p*=0.04), and triglycerides (NLR vs. triglycerides: *r* = −0.126, *p*=0.01).

Then, we checked whether NLR was able to predict the progression from aMCI to AD. To this aim we compared its value in aMCI patients who remained stable in comparison to those progressing to dementia during the follow-up period. We found that NLR was practically equal in these two subgroups of subjects (NLR median value in stable aMCI vs. progressed aMCI: 2.29 vs. 2.28). Of note, only NLR remained significantly lower in stable aMCI (*p*=0.04, while progressed aMCI vs. Controls, *p*=0.07)

### 3.3. Correlation between NLR and Parameters of Cognitive and Functional Status

**Figure 4:**
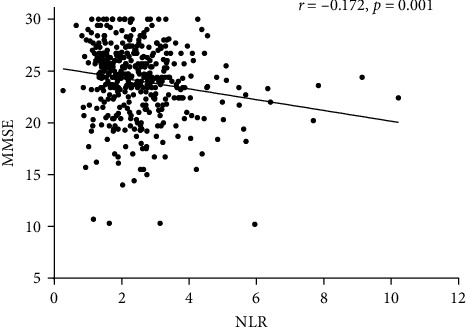
Correlation between NLR and Mini-Mental-State-Examination (MMSE).

In the whole sample, NLR showed an inverse correlation with MMSE score (*r* = −0.172, *p*=0.001, Figure 4), and this was solely driven by aMCI patients (*r* = −0.187, *p*=0.002). On the contrary, the correlation did not reach the statistical significance in the other groups (Controls, *r* = −0.056, *p*=0.710; AD, *r* = −0.072, *p*=−0.236; VAD, *r* = −0.125, *p*=0.201). Notably, within the aMCI group, the association between NLR and MMSE remained significant after adjusting for age, sex, smoking, comorbidities, albumin, and triglycerides (*β* = −0.185, *p*=0.012, data not shown). No significant correlations were found between NLR and index of functional status (BADLs or IADLs).

## 4. Discussion

NLR is a very “attractive” inflammatory marker because it is noninvasive, unexpansive, and easily calculated from the routine blood analysis in many clinical settings [18, 20, 21]. Since systemic inflammation is regarded as a characterizing feature in the pathogenesis of dementia [6, 28], in particular AD, we decided to investigate the potentials of this parameter as diagnostic and predictive biomarker.

The present study dealt with both cross-sectional and longitudinal evaluation of blood NLR.

We found that: (1) high NLR values were significantly and independently associated with AD and aMCI but not with VAD; (2) the NLR was unable to discriminate between aMCI and AD, and between these two and VAD; (3) the NLR was negatively and significantly correlated with the MMSE score (with the association driven by aMCI), the most used neuropsychological test for the screening of cognitive functions; (4) NLR was not a predictor of the possible conversion from aMCI to AD.

Contextualizing our results with the available literature, we noticed an overall consistency with previous reports. To our knowledge, there are only three other studies dealing with the cross-sectional comparison of NLR in AD vs. healthy controls [29, 30, 31]; all found similar, even if more accentuated, differences between the two groups. Of note, mean levels of NLR in diagnostic groups appear to be different from ours (this difference mostly emerged by comparing our Controls to those of the three studies). This could be explained by the different characteristics of the sample, such as age (Controls were from 5 to 8 years younger than ours), race, sex distribution, and comorbidities (no information about their prevalence were punctually reported). Two of these investigations also included MCI patients and, consistent with our findings, found that NLR in this group increased at similar extent of AD [29, 30]. The differences between groups were independent of possible confounding factors in two out of three studies. This might rely on the types of covariates adjusted for in the multivariate analysis. Indeed, the study of Rembach et al. [29] (the largest in this group; *n* = 1,094) was the only one to include also ApoE4, i.e., the most prominent genetic risk factor for AD [32]. However, in contrast with us, they did not consider other important covariates, such as hypertension, CHD, and diabetes, which are widely regarded as risk factors for neurodegenerative diseases as well.

The evaluation of NLR in VAD represents, to the best of our knowledge, a novelty of our study. We observed a modest, not significant, increase in the NLR also in this, suggesting that neutrophils and lymphocytes might be more deeply involved in the pathogenesis of AD compared to other dementia-related diseases. As proof of that, there are many reports showing that the neutrophils proactively participate in the rupture of BBB by upregulating MMPs [6, 33, 34] and largely contribute to the increase of peripheral cytokines observed in AD patients [6]. In turn, these mediators exacerbate the microglia activation and neuroinflammation. Moreover, it has been clearly demonstrated that chemokines and cytokines secreted by overactivated microglia increases neutrophils' survival, thus fueling a vicious and self-promoting cycle that characterizes AD since the early stages [35, 36]. An opposite trend has been shown in this and other studies for lymphocytes in AD [30, 33, 37]. The decreased counts of these cells have been attributed to their substantial recruitment from the periphery to the brain across a compromised BBB [33]. Also, neutrophils migrate to the CNS, but at a lower rate [33]; the mechanisms underlying the difference between the rates of infiltration of the two cell types in the CNS are still unclear.

Differently from us, the same authors did not detect any significant association between NLR and MMSE. Differences in the sample composition could account for this discordancy. Indeed, in our sample, the correlation was driven by aMCI. On the contrary, Rembach's study seemingly included also nonamnestic MCI, which usually has a lower rate of progression to AD [29].

The last finding that is worth discussing is the inability of NLR to predict the conversion of AD: these data are in line with that reported by Rembach et al. [29], showing that NLR did not discriminate subjects who remained stable from those who experienced a cognitive decline over a follow-up of 54 months. Again, their results are difficult to compare with ours, since, in the Australian study, not only MCI but also healthy controls were considered in the longitudinal analysis. Similar considerations can be made as regards another longitudinal study conducted on cognitively healthy individuals from the community-based Framingham Heart Study cohorts (*n* = 1,648 participants and a median follow-up of 5.9 years). This investigation found that a higher baseline NLR was associated with a greater risk of incident dementia [38]. On the basis of these solid results, it is tempting to hypothesize that NLR could be a good predictor of dementia in healthy patients but not in individuals with mild cognitive functions.

Since neuro- and systemic inflammation, along with BBB dysfunction, appears to be a putative pathogenic component of neurodegenerative diseases, alteration of NLR may not be solely related to AD. Interesting examples in this context may be delirium and chronic traumatic encephalopathy (CTE). Delirium is characterized by an acute decline in cognition and attention and is highly prevalent in patients with dementia (with an increase in the risk of adverse outcomes, including death) [39, 40]. A wealth of evidence suggests that delirium may contribute to dementia through several mechanisms, such as inflammation [41, 42]. Indeed, elevation to peripheral markers of inflammation, including NLR, has been found to be related with delirium occurrence [43, 44]. Longitudinal investigations in patients with dementia may help to establish if NLR may be a predictive biomarker for delirium. In a similar fashion, it would be interesting to investigate the clinical usefulness of NLR measurement in patients with CTE. This brain disorder is mostly caused by repeated head injuries and is widely regarded as a risk factor for AD [45]. Neuroinflammation (with overactivation of microglia) has been widely suggested to be a crucial pathological process underlying chronic neurodegeneration following CTE and linking this disease to AD-related neurodegeneration [46, 47].

Some limitations of the study need to be outlined. First, we cannot exclude that some biases or unmeasured confounders (e.g., APOE4) might limit the reliability of our results. However, it is also fair to underline that we adjusted our results for several factors known to be associated with dementia risk (i.e., age, gender, hypertension, CHD, stroke, diabetes, smoking status).

Second, patients were evaluated once; thus, laboratory assessment errors may easily affect the reliability of the results. In the case of the longitudinal analysis of NLR in aMCI, the lack of cognitive and functional trajectory data limits an exhaustive interpretation of our results. Third, the diagnosis of AD was made with well-established criteria based on clinical and imaging techniques (MRI and FDG-PET) but not with cerebrospinal fluid markers. This may have affected the accuracy of the diagnostic process. Finally, the results should be confirmed by large-cohort studies due to the limited number of patients with VAD enrolled into this study. Indeed, we are aware that this low numerosity could have contributed to the negative result obtained with VAD.

## 5. Conclusions

This is the first report showing that NLR is significantly and independently increased in aMCI and AD patients but not other in VAD. We also found that NLR is unable to predict the possible conversion of aMCI to AD. Further research on larger cohorts is warranted to definitely ascertain the application of NLR as an early diagnostic marker for aMCI and AD.

## Figures and Tables

**Table 1:** General characteristics of the sample.

Variables	Controls *n* = 61	aMCI *n* = 214	AD *n* = 103	VAD *n* = 34
Age (years)	77 ± 6	78 ± 5	79 ± 6	80 ± 5^a^
Female gender (%)	59	58	69	73
Formal education (years)	8 (5–11)	5 (5–8)^a^	5 (5–8)^a^	5 (4–11)^a^
Current smoker (%)	10	7	7	—
Comorbidities:				
Hypertension (%)	65	71	57^b^	80^c^
Diabetes (%)	15	18	13	7
CHD (%)	13	8	13	12
Stroke (%)	3	5	2	6
Functional and cognitive status:				
MMSE score (/30)	27 (26–29)	25 (23–27)^a^	21 (19–24)^a, b^	23 (22–26)^a, b^
IADLs (/6)	7 (5–8)	6 (4–8)	4 (3–6)^a, b^	6 (2–7)
BADLs (/8)	6 (5–6)	6 (5–6)	5 (4–6)	5 (4–6)
Clinical chemistry markers:				
Total cholesterol (mg/dL)	202 ± 47	208 ± 42	211 ± 43	215 ± 33
HDL-C (mg/dL)	58 ± 17	61 ± 32	59 ± 15	59 ± 14
LDL-C (mg/dL)	123 ± 42	128 ± 37	130 ± 38	133 ± 27
Tryglicerides (mg/dL)	110 ± 45	110 ± 47	114 ± 58	110 ± 27
Albumin (g/dL)	4.0 (3.9–4.3)	4.1 (3.9–4.2)	4.0 (3.9–4.2)	3.9 (3.7–4.2)
Hb (g/dL)	13.3 ± 1.8	13.1 ± 1.6	13.3 ± 1.8	13.5 ± 1.5
Creatinine (mg/dL)	0.9 ± 0.3	0.9 ± 0.4	0.9 ± 0.2	0.9 ± 0.4
Neutrophiles (n/*µ*L)	3,480 (2,495–4,325)	3,680 (2,930–4,500)	3,820 (2,960–4,570)	3,505 (3,555–4,022)
Lymphocytes (n/*µ*L)	1,719 ± 655	1,691 ± 663	1,688 ± 537	1615 ± 594
NLR	2.0 (1.5–3.1)	2.3 (1.7–3.0)^a^	2.5 (1.8–3.0)^a^	2.2 (1.5–2.8)

^a^ *p* < 0.05 vs. Control; ^b^*p* < 0.05 vs. MCI; ^c^*p* < 0.05 vs. LOAD.

## Data Availability

Data used in this study are available upon request.
